# DAF-18/PTEN signals through AAK-1/AMPK to inhibit MPK-1/MAPK in feedback control of germline stem cell proliferation

**DOI:** 10.1371/journal.pgen.1006738

**Published:** 2017-04-14

**Authors:** Patrick Narbonne, Paul S. Maddox, Jean-Claude Labbé

**Affiliations:** 1 Department of Pathology and Cell Biology, Institut de Recherche en Immunologie et en Cancérologie (IRIC), Université de Montréal, Montréal, Québec, Canada; 2 Department of Biology, University of North Carolina Chapel Hill, Chapel Hill, NC, United States of America; Geisel School of Medicine at Dartmouth, UNITED STATES

## Abstract

Under replete growth conditions, abundant nutrient uptake leads to the systemic activation of insulin/IGF-1 signalling (IIS) and the promotion of stem cell growth/proliferation. Activated IIS can stimulate the ERK/MAPK pathway, the activation of which also supports optimal stem cell proliferation in various systems. Stem cell proliferation rates can further be locally refined to meet the resident tissue’s need for differentiated progeny. We have recently shown that the accumulation of mature oocytes in the *C*. *elegans* germ line, through DAF-18/PTEN, inhibits adult germline stem cell (GSC) proliferation, despite high systemic IIS activation. We show here that this feedback occurs through a novel cryptic signalling pathway that requires PAR-4/LKB1, AAK-1/AMPK and PAR-5/14-3-3 to inhibit the activity of MPK-1/MAPK, antagonize IIS, and inhibit both GSC proliferation and the production of additional oocytes. Interestingly, our results imply that DAF-18/PTEN, through PAR-4/LKB1, can activate AAK-1/AMPK in the absence of apparent energy stress. As all components are conserved, similar signalling cascades may regulate stem cell activities in other organisms and be widely implicated in cancer.

## Author summary

Adult stem cell proliferation rates respond to the needs for their differentiated progeny. For example, the lung stem cells of a smoker will divide more frequently than those of a non-smoker to constantly replenish smoke-damaged tissues. Molecularly, stem cell proliferation rates vary according to growth factor levels, some of which, such as insulin, are regulated systemically. What remains unclear is whether and how a more or less rapid turnover of differentiated cells interferes with growth factor signalling to ultimately set the stem cells’ proliferative rate. We show here that this is accomplished though a novel cryptic signalling pathway that locally antagonizes the effects of insulin through inactivating a more locally-acting growth factor pathway (ERK/MAPK signalling). Interestingly, a ubiquitous protein kinase (AMPK) that has primarily been implicated in energy stress response constitutes an integral part of this feedback mechanism. This suggests that an abundance of differentiated cells is molecularly interpreted as being similar to energy stress at the level of the stem cells, likewise leading to the inhibition of their growth and proliferation.

## Introduction

Stem cells can divide symmetrically to generate more copies of themselves or asymmetrically to produce one stem cell and a daughter cell destined for differentiation. Whether a stem cell will proliferate or differentiate is governed by niche signaling, which locally prevents differentiation [1]. Stem cell proliferation rates on the other hand, are largely controlled by growth factors that act in parallel to niche signaling to stimulate stem cell activity independent of fate [2–9]. Some of these growth factors are systemically controlled, such as insulin/IGF-1 signalling (IIS), which relays nutrient uptake information to cell growth/proliferation, largely through stimulating protein synthesis by activating the mTOR complex [10–12].

In *C*. *elegans* (*Ce*), activated IIS stimulates germline stem cell (GSC) proliferation throughout postembryonic life [2,3,9]. Genetic inactivation of IIS during early larval development leads to entry into a developmentally-arrested stage called dauer in which GSCs are fully quiescent [2,13], while IIS inactivation at later stages of development or adulthood dramatically slows GSC proliferation rates [3,9]. Dauer entry is naturally promoted by food deprivation, which causes energy stress and leads to a down-regulation of IIS and activation the AMP-activated protein kinase AAK (*Ce* AMPK) [14], a master intracellular energy-stress sensor [15]. In this context, the proper establishment of GSC quiescence downstream of insulin receptor inactivation requires DAF-18 (*Ce* PTEN), PAR-4 (*Ce* LKB1; an AMPK-activating kinase [15]), as well as germline AAK activity [2,14].

In addition to being subject to systemic growth factor regulation, the proliferation rates of certain stem cell populations are also affected by the demand for their differentiated progeny. This was first demonstrated in *Drosophila* intestinal stem cells, the proliferation of which can be stimulated by cytokines released by damaged intestinal epithelial cells [5]. In *Drosophila* hematopoietic progenitor cells, proliferation was found to be inhibited by differentiated hemocytes through an adenosine deaminase growth factor A (Adgf-A) signal [16]. In mammals, hair follicle stem cell proliferation is stimulated by their transit-amplifying progeny once these begin producing Sonic hedgehog (Shh) [17]. Interactions between stem cells and their differentiated progeny is thus a conserved phenomenon that is likely to be involved in the regulation of diverse types of stem cells.

Recently, we found that the proliferation rates of the *C*. *elegans* GSCs were negatively influenced by oocyte accumulation in sperm-less or sperm-depleted hermaphrodites [9]. *C*. *elegans* hermaphrodites produce a finite number of sperm during larval development, then switch to oocyte production during adult life, which continues until sperm stores are depleted. Sperm constitutively secrete major sperm proteins (MSPs) that activate cAMP signaling in the proximal somatic gonad. cAMP leads to activation of OMA-1 and OMA-2 in the proximal-most oocyte, triggering oocyte maturation (i.e. the transition between diakinesis and metaphase of meiosis I, which is accompanied by nuclear envelope breakdown, rearrangement of the cortical cytoskeleton, and meiotic spindle assembly) and ovulation [18–21]. Thus, when sperm is absent or depleted, oocytes do not mature and begin to accumulate in the proximal gonad, eventually leading to the inhibition of further oocyte production, as well as the inhibition of GSC proliferation at the distal end [9,19,22]. Indeed, the GSCs of sperm-less or sperm-depleted hermaphrodites enter G2/M quiescence with stochastic transient bouts of proliferation, leading to an overall drastically reduced mitotic index (MI) [9,23].

Feedback inhibition of GSC proliferation by oocyte accumulation requires DAF-18 activity, which acts to locally antagonize systemic IIS information and block GSC proliferation, specifically in the gonad arm that is sperm-depleted and has accumulated oocytes. In this situation, DAF-18 does not block GSC proliferation by directly inhibiting IIS through antagonizing AGE-1 (*Ce* PI3K) activity because the transcription factor that AGE-1 eventually inhibits, DAF-16 (*Ce* FOXO), remains inactivated in sperm-less animals and is dispensable for feedback GSC regulation [9]. Thus, we reasoned that DAF-18 was inhibiting GSC proliferation in parallel to IIS via another, yet unidentified, signalling mechanism. Our work here demonstrates how DAF-18 negatively regulates MPK-1 (*Ce* MAPK) function through activating PAR-4 and AAK-1 (*Ce* AMPK α1-catalytic subunit), in the absence of any apparent stress, to locally couple GSC proliferation rates to the need for their differentiated progeny.

## Results

### PAR-4/LKB1 and AAK-1/AMPK couple GSC proliferation to oocyte needs

**Fig 1:**
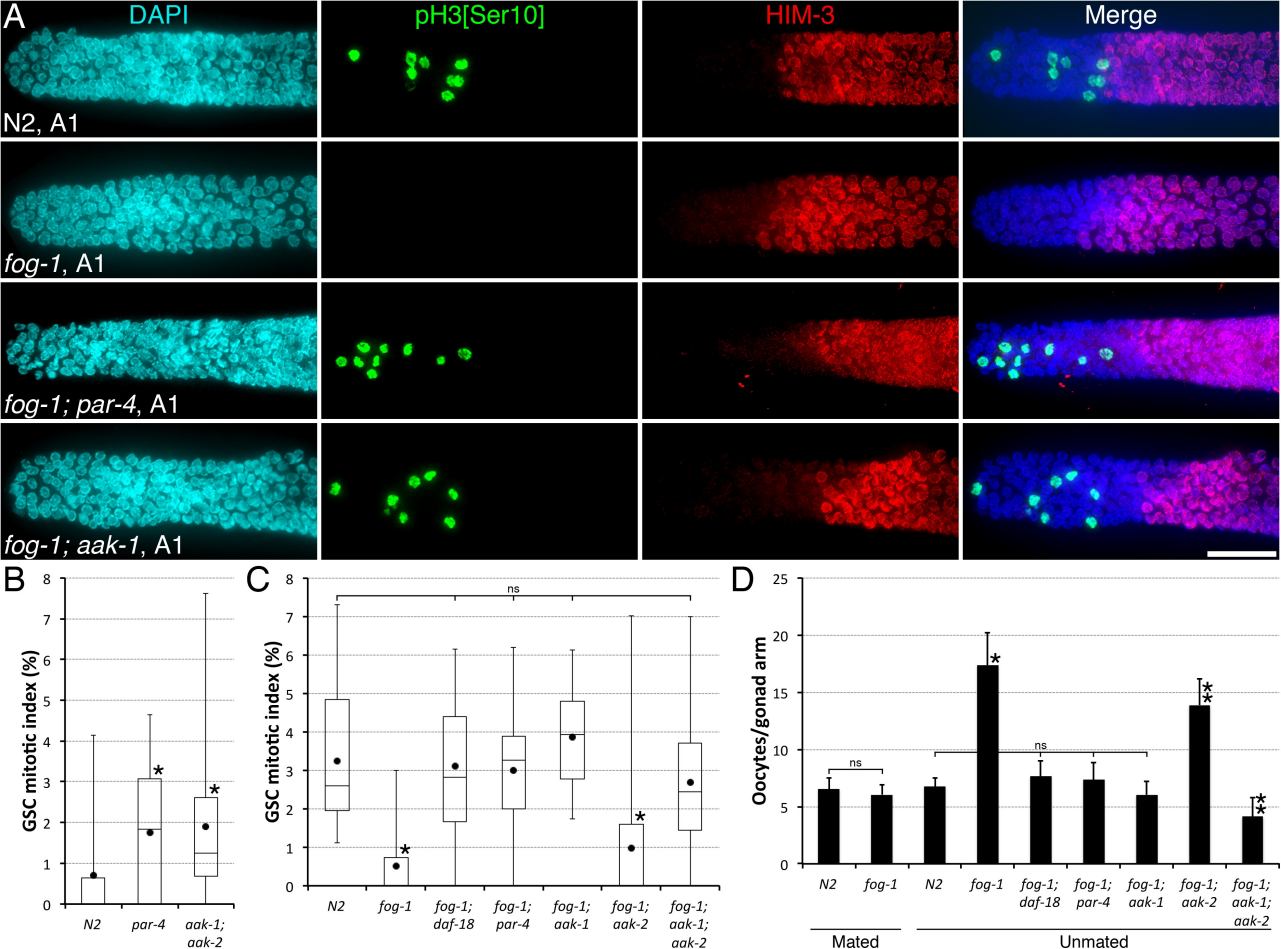
Sperm depletion requires PAR-4 and AAK-1 to promote GSC quiescence. (**A**) Representative distal germ lines dissected from unmated A1 animals of the indicated genotypes, stained with DAPI (DNA; blue), anti-phospho[ser10] histone H3 (M-phase GSCs; green) and anti-HIM-3 (differentiated GSC progeny; red). Distal is to the left. Scale bar: 20 μm. (**B-C)** The GSC MIs of (**B**) A4 sperm-depleted, or (**C**) A1 hermaphrodites of the indicated genotypes were determined. (**B**) Asterisks indicate statistical significance (P<0.05; One-way ANOVA with Holm pairwise comparisons *vs* N2). n, 22–26. (**C**) Asterisks indicate statistical significance (P<0.05; One-way ANOVA with Holm multiple comparisons). n, 15–32. (**B-C**) Dots mark averages, open boxes and brackets represent the whole sample, divided in quartiles. (**D**) Average number (± standard deviation) of diakinesis-stage oocytes per gonad arm in mated and unmated A1 hermaphrodites of the indicated genotypes. An asterisk indicates statistical significance *vs* N2, whereas two asterisks indicate difference *vs* both unmated N2 and unmated *fog-1* (P<0.05; One-way ANOVA with Holm multiple comparisons). n, 24–30. (**A-D**) ns, not significant. Alleles, *fog-1(q785)*; *daf-18(nr2037); par-4(it57); aak-1(tm1944); aak-2(ok524)*.

We first sought to identify the regulators that, together with DAF-18 and independently of DAF-16, promote GSC quiescence in sperm depleted animals. Because PAR-4 and AMPK function with DAF-18 to promote *C*. *elegans* GSC quiescence independently of DAF-16 during dauer development [2], we tested whether PAR-4 and/or AMPK were similarly required to promote GSC quiescence in sperm-depleted, adult day 4 (A4) hermaphrodites. We found that GSC quiescence was not induced in sperm-depleted A4 animals bearing either a strong *par-4* loss-of-function allele [24], or null mutations in both AMPK α-catalytic subunits (*aak-1* and *aak-2*) (Fig 1A and 1B). These mutations similarly disrupted the promotion of GSC quiescence at A1 in sperm-less mutants, such that the GSC MI of *fog-1; par-4* and *fog-1; aak-1; aak-2* animals was indistinguishable from that of wild-type animals or *fog-1; daf-18* double mutants (Fig 1A and 1C). We further noticed that both *fog-1; par-4* double mutants and *fog-1; aak-1; aak-2* triple mutants did not accumulate a large number of unfertilized oocytes in their proximal gonad, a feature of *fog-1* single mutants. Accordingly, the proximal-most oocyte in these animals spontaneously matured in the absence of sperm and was ovulated (Figs 1D and S1), a phenotype also observed in *fog-1; daf-18* mutants [9]. These results indicate that PAR-4 and AMPK are, like DAF-18, required to promote oocyte and GSC quiescence in adult hermaphrodites following sperm depletion.

During dauer entry, both AMPK α-catalytic subunits have a similar and additive effect on the induction of GSC quiescence [2]. We wondered if they acted likewise for the establishment of oocyte and GSC quiescence following sperm depletion in adult animals. Surprisingly, we found that the removal of *aak-1* alone fully recapitulated the loss of *daf-18* or *par-4*, both in terms of quiescent oocyte accumulation and GSC MI regulation (Fig 1A, 1C and 1D). The loss of *aak-2* only had a marginal, yet statistically significant, effect on oocyte accumulation (Fig 1C and 1D). Therefore, AAK-1 is required and sufficient for the induction of both oocyte and GSC quiescence in the absence of sperm. Together with the existing literature [2,9,14], these results suggest that upon sperm depletion, downstream or in parallel to DAF-18, PAR-4 phosphorylates and activates an AAK-1-containing AMPK complex that prevents oocyte maturation. This leads to oocyte accumulation in the proximal gonad arm, which subsequently promotes GSC quiescence in the distal end. The difference in *aak* catalytic subunit requirements between dauer (both *aak-1* and *aak-2*) and adult feedback (*aak-1* only) regulation of GSC proliferation supports the notion that the two responses are mechanistically different.

### AAK-1/AMPK activity is required at multiple steps in feedback control of GSC proliferation

**Fig 2:**
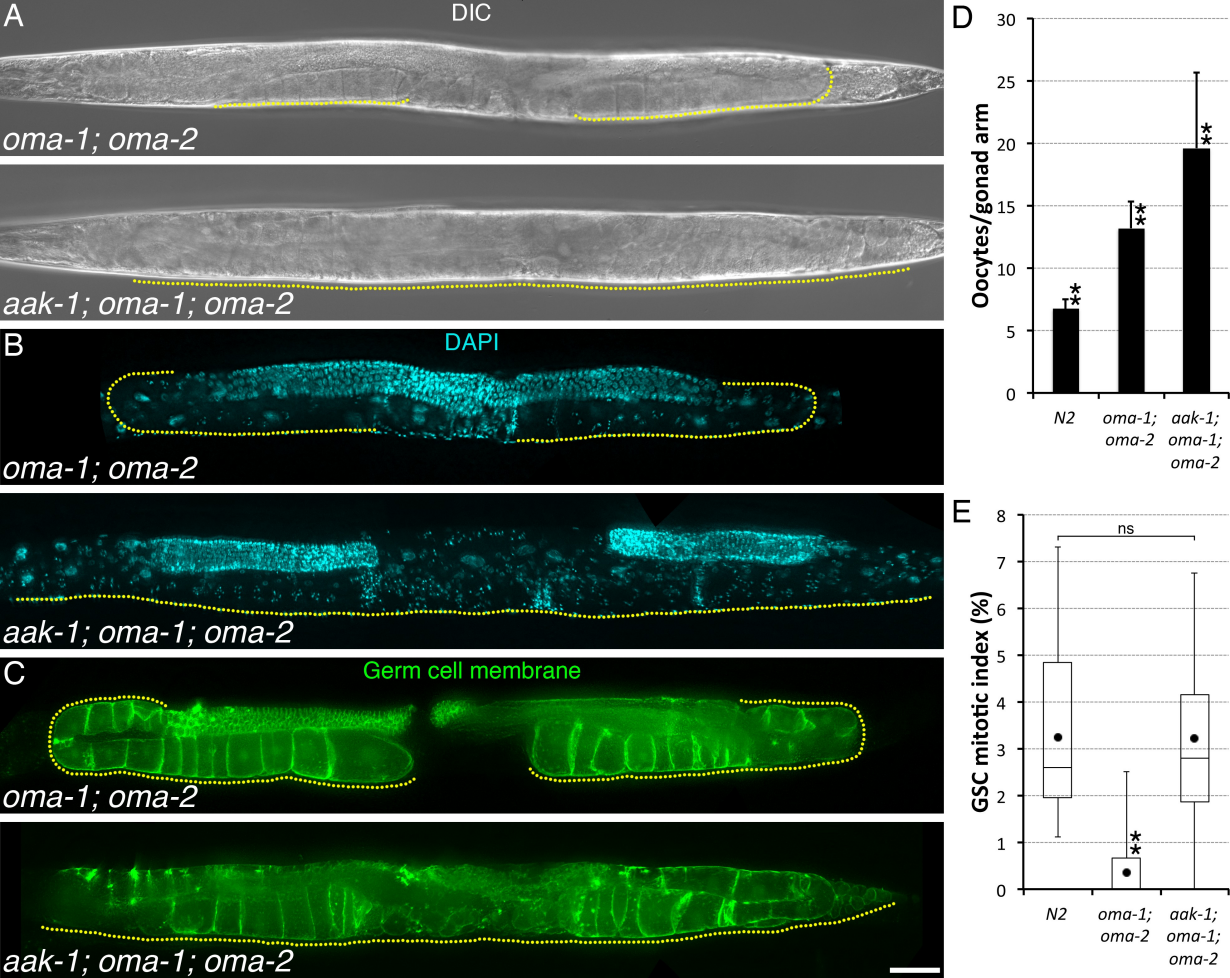
AAK-1 couples GSC proliferation to oocyte accumulation. (**A-C**) Representative (**A-B**) A1 or (**C**) A2 animals of the indicated genotypes imaged using (**A**) Differential Interference Contrast (DIC), (**B**) DAPI (DNA; blue), or (**C**) *mex-5p*::*mNeonGreen*::*PLCδ-PH* (Germ cell membrane; green). A yellow dotted line delineates the area occupied by oocytes. Anterior is to the left and dorsal, up. Scale bar: 50 μm. (**D**) Average number (± standard deviation) of diakinesis-stage oocytes per gonad arm in A1 hermaphrodites of the indicated genotypes. n, 24–28. (**E**) The GSC MIs of A1 hermaphrodites of the indicated genotypes were determined. n, 18–23. Dots mark averages, open boxes and brackets represent the whole sample, divided in quartiles. (**D-E**) Two asterisks indicate statistical significance (P<0.05; One-way ANOVA with Holm multiple comparisons) to all other samples. ns, not significant. (**A-E**) Alleles: *aak-1(tm1944)*, *oma-1(zu405te33)*, *oma-2(te51)*.

**Fig 3:**
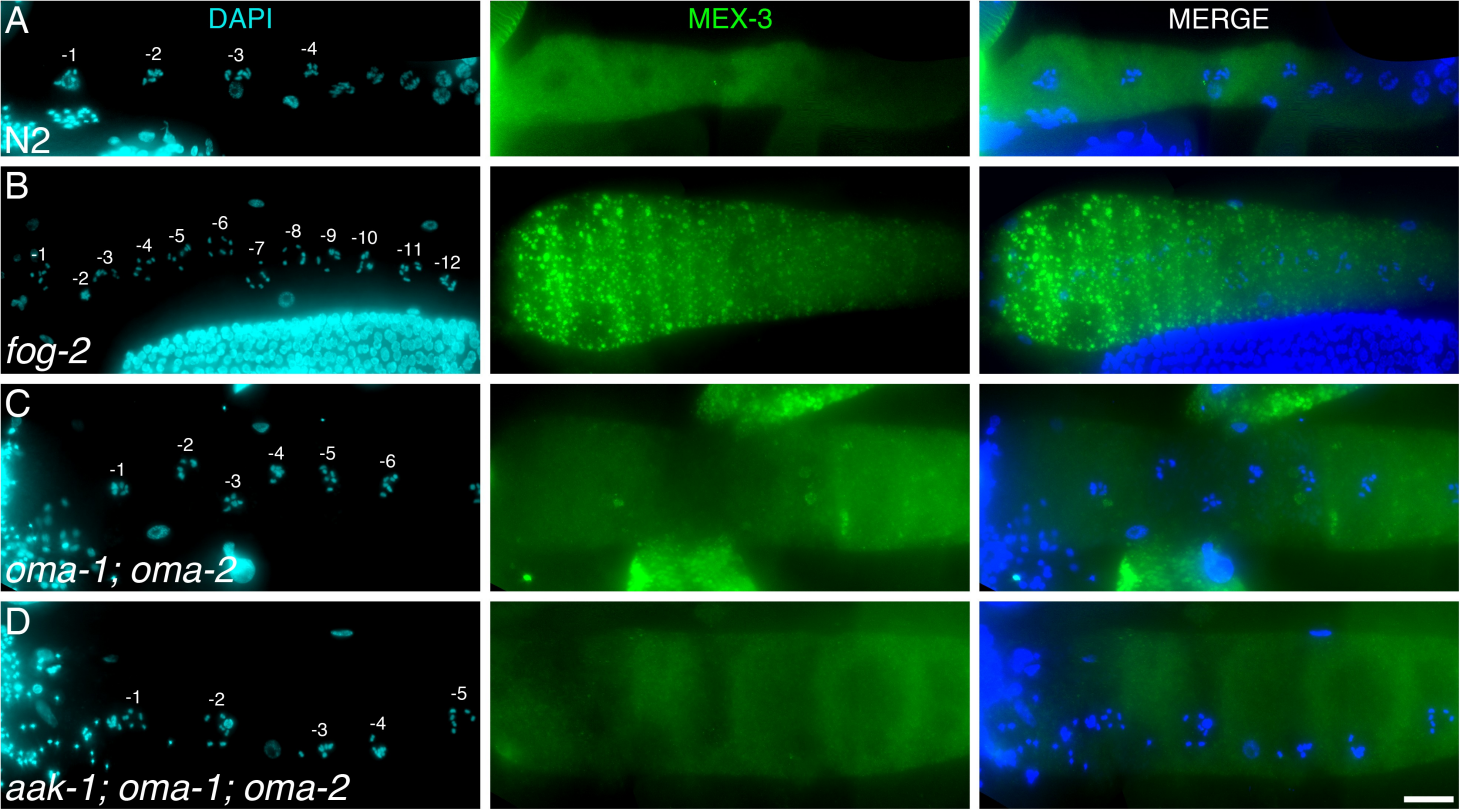
AAK-1 is activated in the absence of oocyte stress. (**A-D**) Representative proximal germ lines dissected from unmated A1 animals of the indicated genotypes, stained with DAPI (DNA; blue) and anti-MEX-3 (a stress granule marker [27]; green). n, 5–7 germ lines were analysed per genotype. Proximal is to the left. Scale bar: 20 μm. Alleles: *aak-1(tm1944)*, *oma-1(zu405te33)*, *fog-2(oz40)*, *oma-2(te51)*.

All of the mutations uncoupling GSC proliferation from sperm availability also prevent oocyte quiescence, retention, and accumulation (Figs 1C, 1D and S1; [9]). Although we had previously suspected that oocyte accumulation triggers the inhibition of GSC proliferation, no condition had allowed us to directly ascertain this. We reasoned that OMA-1/2 were likely to be required for the ectopic oocyte maturation that we had observed in sperm-less or sperm-depleted *aak-1* mutants, thus potentially allowing us to uncouple oocyte accumulation and feedback signalling. In these *oma-1; oma-2* double mutants, sperm is normally produced and activates MSP signaling in the proximal somatic gonad, but the oocytes fail to mature and they accumulate. As in sperm-less *fog-1* or *fog-2* mutants (we did not observe any phenotypic differences between *fog-1* and *fog-2* for any of the measured parameters [9], and thus these two backgrounds were used interchangeably), GSC proliferation is also reduced in *oma-1; oma-2* A1 double mutants (Fig 2A–2E; [9]). *oma-1; oma-2* double mutants thus phenotypically resemble *fog* mutants although they accumulate a smaller number of variably-sized oocytes that do not appear as compressed as those in the germ lines of *fog* mutants [9,18] (also refer to Fig 3A–3C).

We thus generated *aak-1; oma-1; oma-2* triple mutants to see if this would prevent oocyte maturation and induce oocyte accumulation in animals defective for feedback signalling to the GSCs. As expected, we found that oocytes did not undergo spontaneous maturation and accumulated in *aak-1; oma-1; oma-2* triple mutants. Notably, despite the presence of accumulated oocytes, the GSC MI remained elevated at A1 in these animals (Fig 2E), indicating that oocyte accumulation requires AAK-1 activity to promote GSC quiescence. Strikingly however, oocyte production did not slow down and these animals became filled with arrested, diakinesis-stage oocytes within 1–2 days (Fig 2A–2D). Diakenesis-arrested oocytes in *aak-1; oma-1; oma-2* triple mutants accumulated sometimes in multiple, disorganized rows in the gonad arm, filled-up the uterus, and could eventually breach the gonad proper (Fig 2A–2C). Thus, we conclude that AAK-1 is required at three steps for feedback control of GSC proliferation: 1) to permit oocyte accumulation by preventing their spontaneous maturation and ovulation in the absence of sperm/MSP signal, 2) to slow-down oocyte production upon oocyte accumulation, and 3) to inhibit GSC proliferation.

### AAK-1/AMPK blocks GSC proliferation in absence of stress

AMPK is activated by direct binding to 5’-AMP, the concentration of which can dramatically increase during energy stress, but also under many other stressful cellular circumstances that affect ATP usage/production. Significant AMPK activation however requires phosphorylation on a conserved activating residue, which is generally performed by LKB1, a constitutively active kinase [25]. Thus, AMPK has emerged as a master metabolic stress sensor that is turned ON in response to various kinds of stresses and acts to implement a modified energy homeostasis balance [15]. Well-fed sperm-less hermaphrodites (*fog* mutants) have normally activated IIS [9] and a wild-type lifespan [26], suggesting that they do not experience systemic energy stress. However, specific cells could still be subjected to stress during oocyte accumulation, leading to localized, cell-autonomous AMPK activation. Notably, the accumulating unfertilized oocytes in *fog* mutants undergo visible compaction (Fig 3A and 3B; [19,27]) and could be experiencing mechanical stress. Consistent with this, large ribonucleoprotein (RNP) foci (also known as P-bodies or stress granules) form in the compressed and arrested oocytes of *fog* mutants [27]. Similar RNP foci also form in the non-arrested (non-compressed) oocytes of sperm-bearing wild-type animals during heat shock, osmotic stress, or anoxia, suggesting that formation of these RNP foci is a general response of oocytes to stress [27]. Thus, AAK-1 activity could be locally triggered in accumulated oocytes due to a localized stress response and act to block the production of further oocytes and GSC proliferation.

To test this hypothesis, we monitored the localization of MEX-3, a germline protein known to accumulate in RNP granules upon stress [27], in *fog* mutants that were devoid of feedback signalling. We found that RNP foci did not form in the proximal oocytes of *fog-1; daf-18*, *fog-1; par-4* or *fog-1; aak-1* mutants (S2A–S2D Fig). However we could not exclude that this was a consequence of the ectopic oocyte maturation phenotype that we observed in these mutants, which prevents oocyte accumulation (and compression). We therefore monitored MEX-3 localization in the arrested oocytes of *aak-1; oma-1; oma-2* triple mutants, which accumulate but do not undergo oocyte maturation. We found that RNP foci were absent from arrested oocytes of *aak-1; oma-1; oma-2* triple mutants but, to our surprise, they were also absent from the arrested oocytes of *oma-1; oma-2* double mutants (Fig 3A–3D). This suggests that the arrested oocytes of *oma-1; oma-2* double mutants are not experiencing any stress, unlike the seemingly compressed ones of *fog* mutants. Thus, the formation of RNP foci in arrested oocytes is not required for feedback regulation of GSC proliferation. These results suggest that AAK-1 is functioning in this pathway as a signalling kinase rather than as an energy or stress sensor.

### AAK-1/AMPK blocks GSC proliferation through inhibiting MPK-1/MAPK

In both pre-dauer and adult germ lines, DAF-18 is required to suppress GSC proliferation, but can do so independently of DAF-16, the ortholog of FOXO that functions as the main IIS effector in *C*. *elegans* [2,9,26,28]. DAF-18 was reported to impinge on the ERK/MAPK pathway independently of DAF-16 during vulva development and meiotic progression [29,30]. While the *C*. *elegans* ortholog of ERK/MAPK, MPK-1, is present throughout the germ line, its doubly-phosphorylated active form (dpMPK-1) is detectable in two distinct regions of the adult hermaphrodite germ line: late pachytene-stage germ cells (termed “zone I”) and in growing and maturing oocytes (termed “zone II”) [30–32]. Accordingly, *mpk-1* is required for germ cell progression through the pachytene stage and is involved in oocyte growth and maturation [19,31]. Interestingly, it was also reported that null *mpk-1* mutants, which can grow into sterile vulva-less adults, have a small germ line, which is compatible with reduced GSC proliferation [31].

**Fig 4:**
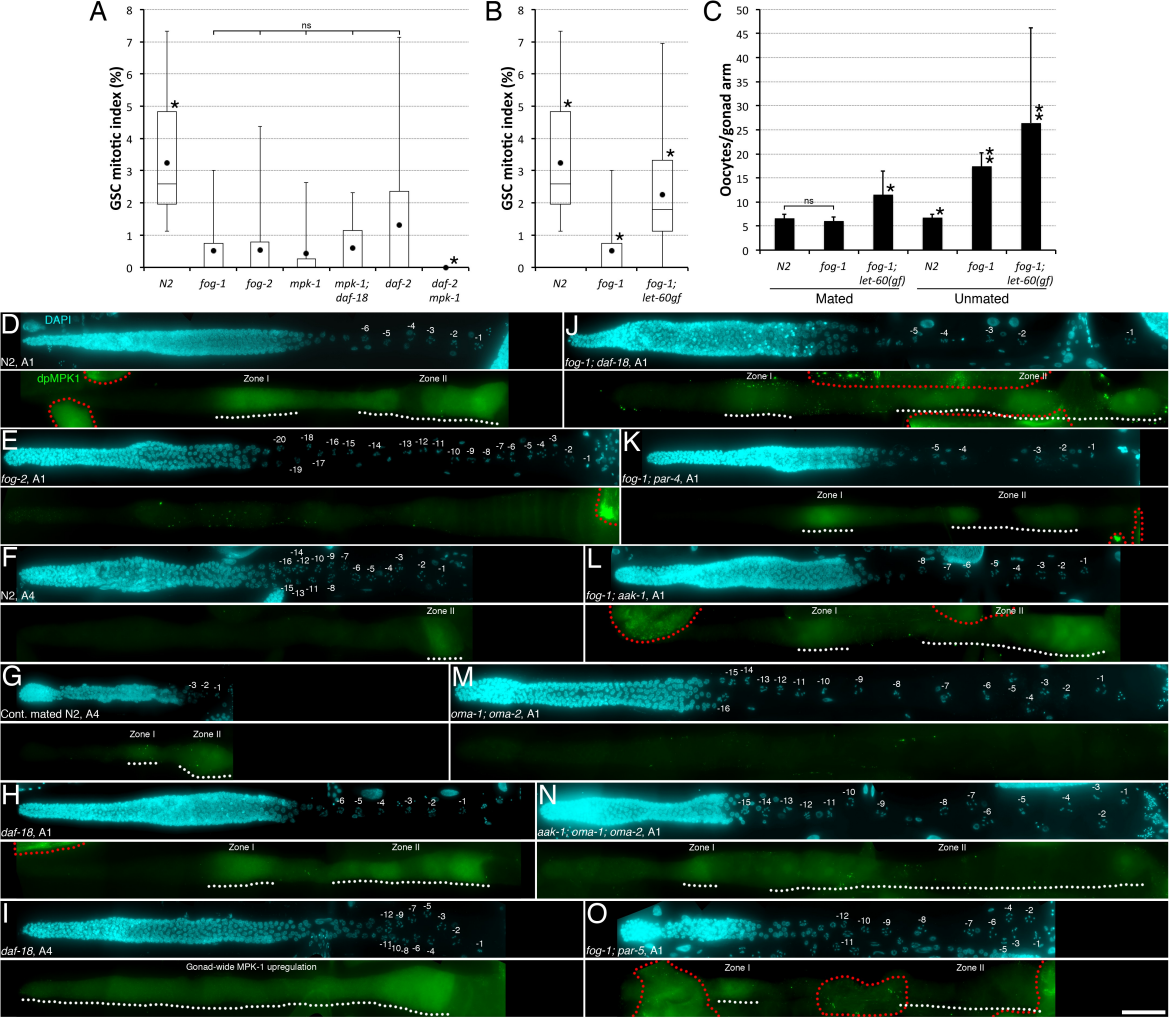
AAK-1 inhibits MPK-1 downstream of oocyte accumulation to promote GSC quiescence. (**A-B**) The GSC MIs of A1 hermaphrodites of the indicated genotypes were determined. (**A**) Asterisks indicate statistical significance (P<0.05; Kruskal-Wallis with Mann-Whitney multiple comparisons) to all other samples. n, 18–30. (**B**) Asterisks indicate statistical significance (P<0.05; ANOVA with Holm multiple comparisons) to all other samples. n, 18–29. (**A-B**) Dots mark averages, open boxes and brackets represent the whole sample, divided in quartiles. (**C**) Average number (± standard deviation) of diakinesis-stage oocytes per gonad arm in mated and unmated A1 hermaphrodites of the indicated genotypes. Asterisks indicate statistical significance (P<0.05; ANOVA with Tukey HSD multiple comparisons) to all other samples within the same condition. Two asterisks indicate additional statistical significance (P<0.05; two-tailed t-test) *vs* mated animals of the same genotype. n, 24–32 (**A-C**). ns, not significant. (**D-O**) Representative germ lines extruded from unmated or continually mated animals of the indicated genotypes and ages, stained with DAPI (DNA; cyan) and a monoclonal mouse anti-MAPKYT that labels dpMPK-1 (green) are shown. White dotted lines underline zones I-II where elevated cytoplasmic dpMPK-1 is apparent. Zone I corresponds to late pachytene germ cells, and zone II, to diakinesis-stage oocytes [30]. Red dotted lines delineate areas of unspecific signal. Distal is to the left. Oocytes are labelled with negative values from the proximal end. n, 7–15 germ lines were analyzed for each condition; fluorescence intensity profiles across zone I are shown in S4 Fig and other representative examples are shown in S5 Fig. dpMPK-1 signal were identical in A1 *fog-1* and *fog-2* germlines (S5 Fig). Scale bar, 50 μm. (**A-O**) Alleles, *fog-1(q785)*, *aak-1(tm1944)*, *mpk-1(ga117)*, *daf-2(e1370)*, *daf-18(ok480)* (panel A), *daf-18(nr2037)* (panels H-J), *let-60(ga89)gf*, *par-4(it57)*, *oma-1(zu405te33)*, *fog-2(oz40)*, *oma-2(te51)*, *par-5(it55)*. *par-5(it55)* mutants are also homozygous for *unc-22(e66)*.

We reasoned that if DAF-18 activity acts to down-regulate MPK-1 following sperm depletion, DAF-18 could potentially block GSC proliferation as well as germ cell progression through the pachytene stage. To test this hypothesis, we first verified that *mpk-1* was indeed required for optimal GSC proliferation. As expected, we found that the GSC MI of A1 null *mpk-1* mutant hermaphrodites was significantly lower than that of wild-type animals, and similar to that of A1 *fog-1* or *fog-2* mutants (Fig 4A). Thus, *mpk-1* activity is required to maintain a high level of adult GSC proliferation. It was previously reported that dpMPK-1 levels decrease in the germ line of sperm-depleted animals, first in the proximal oocytes, and subsequently, but only after oocytes have accumulated, in pachytene-stage germ cells [19,30,31]. To test if the inactivation of MPK-1 is required for sperm depletion to promote GSC quiescence, we up-regulated MPK-1 signalling in *fog-1* mutants using a conditional gain-of-function (*gf*) allele in the gene encoding the *C*. *elegans* Ras ortholog, *let-60* [33,34]. Under restrictive conditions, A1 *fog-1; let-60(gf)* double mutants had a GSC MI that was intermediate to that of A1 *fog-1* mutants and A1 wild-type animals (Fig 4B). Consistent with a previously-reported phenotype of *let-60(gf)* mutants [31], *fog-1; let-60(gf)* double mutants accumulated a greater number of smaller oocytes (Fig 4C). These results indicate that hyperactivation of Ras can alleviate GSC quiescence upon oocyte accumulation and suggest that inactivation of MPK-1 is required for oocyte accumulation to block GSC proliferation.

If *daf-18* and *mpk-1* were acting as part of the same signalling pathway during feedback control of GSC proliferation, we would expect the effect of the two mutations not to add up, but that one (acting downstream) would cancel the effect of the other. We thus asked if a null mutation in *daf-18* modified the low GSC MI phenotype of *mpk-1* null mutants. We found that A1 *mpk-1; daf-18* double null mutants had a low GSC MI that was indistinguishable from that of *mpk-1* single mutants (Fig 4A), indicating that MPK-1 acts downstream of (or in parallel to) DAF-18 to maintain elevated GSC proliferative activity. To confirm that this DAF-18-to-MPK-1 axis of signalling that controls GSC proliferation is acting in parallel to IIS, we tested whether IIS inhibition would be additive to the loss of MPK-1 activity. Strikingly, we found that the GSCs of A1 *daf-2 mpk-1* double mutants do not divide and have a null MI (Fig 4A), indicating that adult GSC proliferation absolutely requires at least one of these two growth factors and that they largely act in parallel.

To determine whether the stimulatory effect that the DAF-18-to-MPK-1 axis of signalling has on GSCs depends on IIS, we measured the GSC MI at A1 in both *daf-2; par-4* and *daf-2; let-60(gf)* double mutants. We found that these two mutations, unlike a mutation in *daf-18* [9], did not significantly restore the GSC MI in *daf-2* mutants (S3 Fig), indicating that the DAF-18-to-MPK-1 axis of signalling does not directly inhibit IIS in wild-type adults, but specifically antagonizes activated IIS in sperm-less hermaphrodites. While loss of either *daf-18* or *par-4* impairs the establishment of GSC quiescence during dauer development [2], we found that the ectopic activation of MPK-1 signalling in *let-60(gf)* dauer animals had no significant effect on GSCs (S3 Fig). These results suggest that control of GSC proliferation during dauer formation does not require MPK-1 inhibition, and that MPK-1 inhibition is thus specifically required for feedback control in adults. Altogether, these results suggest that upon sperm depletion, DAF-18 inhibits MPK-1 activity through PAR-4 and AAK-1 to antagonize IIS independently of DAF-16, and block GSC proliferation in response to oocyte accumulation.

To further test this hypothesis, we evaluated the levels MPK-1 phosphorylation on activating residues when oocytes accumulate in the absence of sperm. In wild-type animals, dpMPK-1 levels peak in the -1 oocyte, and the high level of proximal staining is dependent on MSP signalling [18,19]. In young adult *oma-1; oma-2* double mutants producing their first few oocytes, dpMPK-1 is transiently detectable in the first oocytes, indicating that they do receive the sperm MSP signal, however it is not maintained thereafter [18]. Thus, MPK-1 activation in zone II requires MSP signalling, but maintenance of MPK-1 activity is further dependent on oocyte maturation and/or the presence of OMA proteins. As previously reported, we found that dpMPK-1 levels in zones I-II decreased to background upon oocyte accumulation in A1 *fog-1* and *fog-2* mutant hermaphrodites, in *oma-1; oma-2* double mutants, as well as in unmated, sperm-depleted A4 wild-type hermaphrodites (Figs 4D–4F, 4M, S4 and S5; [18,19,31]). In contrast, dpMPK-1 levels remained elevated in zones I-II in continually mated A4 wild-type hermaphrodites (Figs 4G, S4 and S5). We found that the decrease of dpMPK-1 levels in zones I-II of Fog and Oma animals requires DAF-18, PAR-4 and AAK-1 activities (Figs 4H–4N, S4 and S5). Of note, loss of DAF-18 led to MPK-1 upregulation ectopically throughout the gonad in A4 animals (Figs 4I, S4 and S5; [30]). These results, in combination with the existing literature, suggest that when sperm is depleted, MPK-1 is first inactivated in zone II of the germ line [19,20,31], and that oocyte accumulation then triggers a feedback signal that further inhibits MPK-1 more distally in zone I. Oocyte accumulation-mediated MPK-1 inactivation, first in zone II and then in zone I, would then be required to block adult GSC proliferation, either directly or indirectly.

To gain insight into where MPK-1 inactivation may be required to inhibit GSC proliferation, we removed *mpk-1* activity from *gld-3 nos-3* double mutant animals, in which GSC differentiation is completely blocked, preventing the formation of zones I-II altogether, and leading to the formation of germline tumours [35,36]. We found that the lack of *mpk-1* activity suppressed the growth of germline tumours in *gld-3 nos-3* animals (S5 Fig), indicating that during development, MPK-1 functions either cell autonomously in the GSCs and/or non-cell autonomously in the soma to promote GSC proliferation. Interestingly, we did not measure a significant difference in the GSC MI between *gld-3 nos-3* and *gld-3 nos-3; mpk-1* A1 animals (S6 Fig). This suggests that during adult life, MPK-1 activation in differentiated germ cells is required to promote GSC proliferation in *mpk-1(+)* animals. We conclude that upon sperm depletion, DAF-18, PAR-4 and AAK-1 are required to prevent OMA-1 and OMA-2 activation in the absence of MSP signalling and thus permit the accumulation of quiescent oocytes in the proximal gonad. Upon oocyte accumulation, the activation of DAF-18, PAR-4 and AAK-1 induces the inhibition of MPK-1 activity in zones I-II of the germline, which antagonizes IIS signals and induces GSC quiescence while also blocking meiotic progression and the production of further oocytes.

### PAR-5/14-3-3 is required to inhibit MPK-1/MAPK and block GSC proliferation in response to sperm depletion

**Fig 5:**
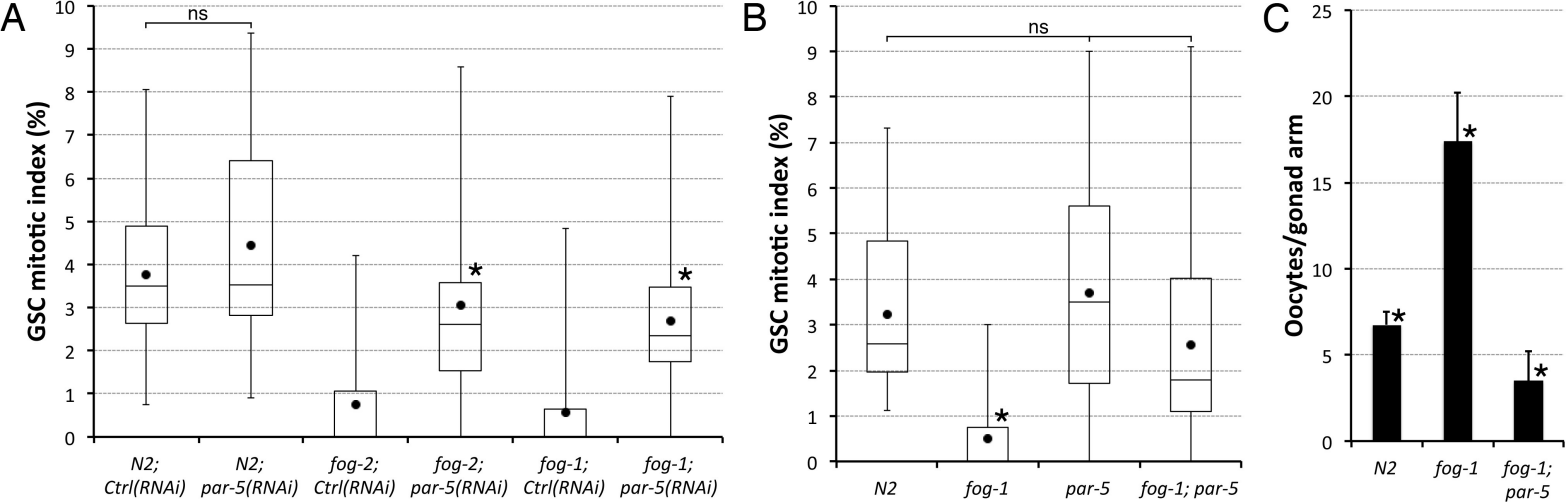
Sperm depletion requires PAR-5 to promote GSC quiescence. (**A-B**) The GSC MIs of A1 hermaphrodites of the indicated genotypes/conditions were determined. Dots mark averages, open boxes and brackets represent the whole sample, divided in quartiles. Ctrl, control RNAi vector L4440. ns, not significant. (**A**) Asterisks indicate statistical significance (P<0.05; Kruskal-Wallis with Mann-Whitney pairwise comparisons to respective RNAi controls). n, 22–28. (**B**) An asterisk indicates statistical significance (P<0.05; ANOVA with Tukey HSD multiple comparisons) to all other samples. n, 18–24. (**C**) Average number (± standard deviation) of diakinesis-stage oocytes per gonad arm in A1 hermaphrodites of the indicated genotypes. Asterisks indicate statistical significance (P<0.05; ANOVA with Tukey HSD multiple comparisons) to all other samples. n, 14–24. (**A-C**) Alleles, *fog-1(q785)*, *par-5(it55)*, *fog-2(oz40)*. *par-5(it55)* mutants are also homozygous for *unc-22(e66)*.

The consensus recognition motif for AMPK is well defined and conserved [15,25], and there is an overlap between a subset of potential AMPK consensus phosphorylation sites and that of the signalling adaptor protein 14-3-3 [25,37,38]. The functional relevance of this motif overlap has been previously demonstrated. For example, phosphorylation of Raptor by AMPK in cultured mammalian cells has been demonstrated to induce 14-3-3 binding, which in turns leads to Raptor inactivation [37]. Interestingly, PAR-5 (the *C*. *elegans* germline-expressed ortholog of 14-3-3 [39]) was found to be required for the maintenance of oocyte quiescence in an RNAi screen designed to isolate regulators of oocyte maturation [40]. This raised the possibility that following sperm depletion, binding between PAR-5 and AAK-1-phosphorylated MPK-1 regulators could lead to the inactivation of this pathway to couple both oocyte and GSC quiescence to sperm availability. To test this hypothesis, we first asked if *par-5* was required to couple GSC proliferation with oocyte needs in *C*. *elegans* adults. We found that RNAi inactivation of *par-5* in either *fog-1* or *fog-2* mutants prevented feedback inhibition of GSC proliferation (Fig 5A). Similarly, GSC proliferation was not significantly inhibited in A1 *fog-1; par-5* double mutants (Fig 5B). Thus, sperm depletion requires PAR-5 to inhibit GSC proliferation. As was the case for *daf-18*, *par-4* and *aak-1*, inactivation of *par-5* caused spontaneous oocyte maturation and ovulation in *fog-1* mutants, preventing their accumulation (Fig 5C) and stress granule formation (S2 Fig). This suggests that all four genes act in the same signalling pathway to retroactively couple GSC proliferation to oocyte needs.

To determine if PAR-5 is required to inhibit MPK-1 activity in zone I following sperm depletion, we measured the levels of dpMPK-1 in A1 *fog-1; par-5* loss-of-function mutants. As for *fog-1; daf-18*, *fog-1; par-4* and *fog-1; aak-1* mutants, we found that the germ line of A1 *fog-1; par-5* double mutants had increased levels of dpMPK-1 in zones I-II relative to the background, albeit levels in zone II were variable between gonads (Figs 4O, S4 and S5). Thus, PAR-5 is required to inactivate MPK-1 in late-pachytene stage germ cells following sperm depletion. Given these results and the functional overlap between the AMPK consensus site and that of 14-3-3, these results suggest that PAR-5 may act to mediate the effects of AMPK phosphorylation to ultimately inhibit MPK-1 activity in the pachytene area of the germ line to promote GSC quiescence.

## Discussion

**Fig 6:**
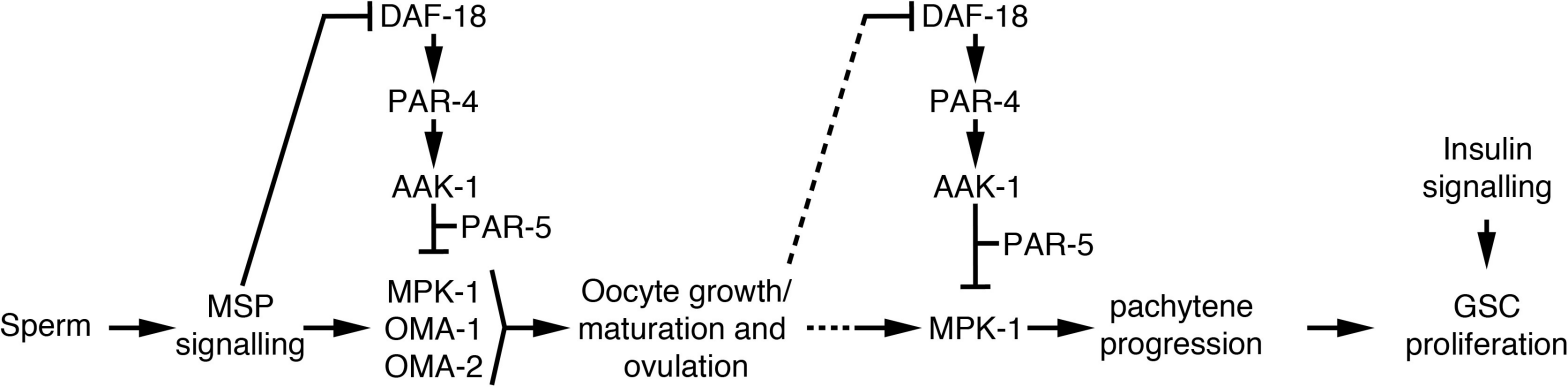
Model for feedback control of GSC proliferation. Arrows represent activation and bars represent inhibition. The dotted lines represent the delay between the coupling of oocyte maturation/ovulation and the regulation of MPK-1 in zone I due to the time required for oocyte accumulation or its relief. Note that MPK-1 activity could regulate GSC proliferation indirectly through affecting pachytene progression (as represented), but also potentially through a parallel mechanism. Also note that we placed *par-5* downstream of *aak-1*, but this is purely due to the overlap between the two proteins consensus target motif.

Our results uncover a novel signalling pathway that links, in two sequential steps, oocyte needs to the regulation of GSC proliferation in *C*. *elegans* adult hermaphrodites (Fig 6). We previously demonstrated that DAF-18 was required to inhibit GSC proliferation downstream of sperm depletion, and that in the absence of sperm in these animals, unfertilized oocytes spontaneously activated and were ovulated [9]. Here, we demonstrate that the lack of PAR-4, AAK-1 or PAR-5 causes a similar phenotype, disrupting oocyte quiescence and feedback regulation of GSC proliferation. Preventing oocyte activation in a feedback-defective mutant also revealed an additional coupling step, this time between oocyte maturation and oocyte production. In *aak-1; oma-1; oma-2* triple mutants, loss of OMA-1 and OMA-2 prevents oocyte maturation, triggering their accumulation, while the loss of AAK-1 blocks feedback signalling to the rest of the germ line. In this case, neither GSC proliferation nor oocyte maturation is blocked and we observe hyper-accumulation of oocytes. Thus, in feedback-defective animals, the accumulation of diakinesis-arrested oocytes cannot block GSC proliferation or oocyte production. This rules out the possibility that oocyte accumulation is physically sufficient to promote GSC quiescence, in which case the accumulated oocytes in *aak-1; oma-1; oma-2* triple mutants would have been expected to promote GSC quiescence.

We favor the possibility that oocyte accumulation indirectly promotes GSC quiescence through first causing an arrest in oocyte production, by blocking pachytene progression upstream in the germ line. Stalling of germ cell progression through meiosis would concomitantly inhibit GSC proliferation further upstream, in a chain of interdependent retrograde events, as we previously proposed [9]. In this scenario, AAK-1 would act as the gear that couples oocyte accumulation to the arrest of oocyte production (and other feedback steps). The newly demonstrated involvement of *mpk-1* in feedback GSC regulation fits well such a mode of operation. Indeed, our results (combined with those of others [9,19,30,40]) are consistent with a model in which sperm depletion blocks oocyte maturation and ovulation, causing their accumulation. After oocytes have accumulated up to a certain point, levels of dpMPK-1 in the pachytene zone of the germ line drop, blocking meiotic progression and the production of additional oocytes. Our results indicate that this inactivation of MPK-1 in pachytene-stage germ cells following oocyte accumulation requires DAF-18, PAR-4, AAK-1 and PAR-5 activities, and that ectopic activation of *mpk-1* signalling prevents oocyte accumulation from blocking GSC proliferation. Lastly, we found that inactivation of *mpk-1* suppressed the effects of a *daf-18* null allele on GSC proliferation, indicating that MPK-1 functions downstream of, or in parallel to, DAF-18. Thus, we propose that following oocyte accumulation, DAF-18 activity leads to MPK-1 downregulation in pachytene-stage germ cells through activation of PAR-4 and AAK-1 (PAR-4 can directly phosphorylate and activate AAK-1/AMPK [14,15]), followed by AAK-1 phosphorylation of yet unidentified targets and their recognition by PAR-5, to inhibit MPK-1 signalling (Fig 6). How this inactivation of MPK-1 exactly inhibits GSC proliferation remains to be elucidated and is a focus for future investigation. However, our analysis of GSC proliferation in tumorous animals that lack differentiated germ cells imply that *mpk-1* activity may promote GSC proliferation partly in the soma and/or GSCs, as well as in differentiated germ cells in the adult, possibly in zone I. Despite their anticipated germline requirement, the precise site(s) of PAR-4/LKB1 and AAK-1/AMPK action in feedback control of GSC proliferation remains to be determined.

Our results indicate that the regulation of larval and adult GSC proliferation is mechanistically distinct. Feedback regulation of GSC proliferation by oocyte accumulation requires PAR-4/LKB1 and AAK-1/AMPK. These genes are also required for inhibition of GSC proliferation during dauer development but in this context they function downstream of insulin and/or TGF-β signals [2]. Our work reveals that PAR-4 and AAK-1 control GSC proliferation upon oocyte accumulation in the absence of changes in IIS during adulthood. The molecular context of feedback regulation of GSC proliferation by oocyte accumulation is thus mechanistically distinct from inhibition of GSC proliferation during dauer development, despite some overlap in gene requirements. Our results suggest that the inhibition of MPK-1 signalling in differentiated germ cells by AAK-1 may be an adult-specific mechanism through which GSC proliferation is controlled. While this mechanism must permit localized regulation of GSC proliferation, it appears to function only in animals that have activated IIS, perhaps because the inhibition of IIS also inactivates MPK-1 in germline zone I [30].

The involvement of AAK-1/AMPK in this feedback signal is particularly interesting because AMPK has been historically predominantly implicated in cellular stress response, typically inhibiting cell growth and proliferation in response to nutrient stress [2,15,25]. Hormones that increase intracellular Ca^2+^ can however activate AMPK in the absence of stress via phosphorylation by the calmodulin-dependent protein kinase CaMKKβ, but this is independent of PAR-4/LKB1 [25,41–43]. To our knowledge, the feedback mechanism that we herein report is thus the first documented occurrence of AMPK activation in a PAR-4/LKB1-dependent context in the absence of any apparent stress, but in response to a specific signal. Although we did not provide molecular evidence of AAK-1/AMPK activation, we assume it is the case due to the concomitant requirement for the upstream AMPK activating kinase PAR-4/LKB1. As for the regulation of lipid reserves in dauer larvae [44], only one of the two catalytic subunit isoforms is sufficient to fulfill this function. Thus, an emerging role of AAK-1/AMPK is its function as a signalling molecule downstream of DAF-18/PTEN and PAR-4/LKB1 to inhibit stem cell proliferation through PAR-5/14-3-3-dependent MPK-1/MAPK inhibition.

Cancer is emerging as being a disease of the stem cells. For instance, cancer incidence was proposed to be related to the frequency of stem cell divisions [45], suggesting it is the stem cells themselves that can turn ill after going through a number of divisions and become tumorigenic. Understanding how stem cell division rates are regulated *in vivo* is thus immediately relevant to cancer biology. In this regard, our results reveal that some tumour suppressor genes, such as PTEN and LKB1, may prevent cancer by limiting stem cell divisions when these divisions are not needed. Indeed, defective feedback inhibition of intestinal stem cells may well underlie the growth of the related benign hamartomas in Cowden’s and Peutz-Jegher’s syndromes, which are caused in humans by germline mutations in PTEN and LKB1, respectively [46–48]. As such, we predict that the mechanisms we have identified in feedback GSC regulation in *C*. *elegans* may underlie cancer development in humans.

## Materials and methods

### *C*. *elegans* genetics

Animals were maintained at 15°C on standard NGM plates and fed *E*. *coli* bacteria (OP50) unless otherwise indicated [49]. The Bristol isolate (N2) was used as wild-type throughout. The following alleles, deficiencies and transgenes were used. LGI: *fog-1(q785)*. LGII: *gld-3(q730)*, *nos-3(q650)*, *cpIs42[mex-5p*::*mNeonGreen*::*PLCδ-PH*::*tbb-2 3’UTR + unc-119(+)]* (generously provided by Drs Dan Dickinson and Bob Goldstein [50]). LGIII: *daf-2(e1370)*, *aak-1(tm1944)*, *mpk-1(ga117)*. LGIV: *daf-18(nr2037*, *ok480)*, *oma-1(zu405te33)*, *par-5(it55)*, *unc-22(e66)*, *let-60(ga89)gf*. LGV: *fog-2(oz40)*, *par-4(it57)*, *oma-2(te51)*, *qIs56[lag-2p*::*GFP; unc-119(+)]*. LGX: *aak-2(ok524)*. The following rearrangements were used. *qC1[dpy-19(e1259) glp-1(q339) qIs26[rol-6(su1006)gf; lag-2p*::*GFP]]III*, *hT2[bli-4(e937) let-*?*(q782) qIs48](I;III)*, *mIn1 [mIs14 dpy-10(e128)] II*, *nT1[unc-*?*(n754) let-*?*] (IV;V)*. RNAi was induced by feeding from the L1 stage as previously described [51].

### GSC mitotic index evaluation

GSC mitotic indexes were evaluated as previously described [9], by transferring late-L4 stage animals from 15°C to a new plate at 25°C and allowing them to grow for an additional 24 hours. A1 animals were then harvested and their gonads were dissected and stained as described below. *4'6-diamidino-2-phenylindole* (DAPI) was used to highlight germ nuclei. Anti-HIM-3 antibodies were used to mark (and exclude) differentiated GSC progeny (HIM-3 positive), and anti-phospho[ser10]-histone H3, to mark M-phase nuclei. Undifferentiated germ nuclei counting in 3 dimensions was partially automated as described [11], with an ImageJ plugin developed by Dr Jane Hubbard’s laboratory. For every genotype/condition for which the MI was previously published (unmated A1: N2, *fog-1*, *fog-1; daf-18*, *oma-1; oma-2*, *daf-2*; [9]), we did not observe a significant difference between the older and newer MI datasets (P>0.05; two-tailed t-test).

### Diakinesis oocytes counts

A1 animals were generated as above, fixed in Carnoy’s solution and stained with DAPI as previously described [2]. The number of diakinesis-stage oocytes (having a least one condensed bivalent chromosome [52]) per gonad arm was then determined. In some backgrounds, the chromosomes in one to a few of the proximal-most oocytes sometimes appeared all condensed together and these were included in the analysis. Endomitotic oocytes were however excluded.

### Staining

Whole animals were fixed in Carnoy’s solution and stained with DAPI [2]. For germline immunofluorescence, gonads were dissected out of the animal in a drop of PBS on a cover slip, which was then placed against a poly-L-lysine coated slide, submitted to a freeze-crack and stained as previously described [9,53]. Primary mouse monoclonal anti-phospho[ser10]-histone H3 (1:200, Cell Signaling #9706), mouse monoclonal anti-double-phospho[Thr202/Tyr204]MAPK (anti-MAPKYT) (1:100, Cell Signaling #9106), rabbit polyclonal anti-HIM-3 (1:500, a generous gift from Dr Monique Zetka) [53], mouse monoclonal anti-MEX-3 (1:100, a generous gift from Dr Jim Priess) [27], and secondary A488-conjugated goat anti-mouse, or A546-conjugated goat anti-rabbit antibodies (both at 1:500, Invitrogen) were used. DAPI was used as a counterstain.

### Image acquisition and processing

Images were acquired at a 0.75 μm (Figs 1 and 2B) or 1 μm (Figs 2C, 3, 4, S1, S2, S5 and S6) intervals using a 20x (whole-worm) or a 60x (germ line) objective on either of two DeltaVision microscopes, deconvolved (whole-worm and distal germ lines), maximally-projected, stitched (whole-worm and whole germ lines), and thresholded using ImageJ. All images are maximal projections, except DIC images, which show a single focal plane. For display and ease of comparison purposes, whole-worms and germ lines were computationally straightened using ImageJ, except for S5 Fig. Fluorescence intensity profiles were obtained and processed essentially as previously described [30] using ImageJ, except that they were obtained from stitched maximal projections instead of single focal planes, and that the background from each stitched image was individually subtracted. Images were reconstructed by stitching multiple, overlapping acquisitions of the same sample using the “Grid/collection stitching with sequential images” plugin with default parameters in ImageJ [54]. Samples in Figs 4 and S4D–S4I, S4K and in S4J, S4L–S4O were acquired on a different microscope/setting that gave rise to a lower signal-to-noise ratio. To partly (and conservatively) circumvent this issue, we defined the background germline signal as the highest signal intensity observed with the better signal-to-noise microscope/setting in animals without dpMPK-1 signal in zones I-II (e.g. *fog-2*, A1).

## Supporting information

S1 Fig Spontaneous oocyte maturation and ovulation in *daf-18*, *par-4* and AMPK mutants. (**A-E**) Representative A1 animals of the indicated genotypes were stained with DAPI. A white dotted line underlines the region occupied by arrested unfertilized oocytes. A red dotted line marks the uterine region, occupied by (**A**) embryos, (**C-E**) spontaneously matured endomitotic oocytes, or (**B**) empty. Anterior is to the left and dorsal, is up. Alleles, *fog-1(q185)*, *daf-18(nr2037)*, *par-4(it57)*, *aak-1(tm1944)*, *aak-2(ok524)*. Scale bar, 100 μM.
(TIF)

S2 Fig Loss-of-function mutations in *daf-18*, *par-4*, *aak-1* or *par-5* block the formation of MEX-3 granules in the absence of sperm. (**A-E**) Representative A1 proximal gonads extruded from animals of the indicated genotypes, stained with DAPI (cyan) and anti-MEX-3 (green), are shown. Diakinesis oocytes are negatively labeled from the proximal-most one. Positive numbers indicate ovulated oocytes (*in utero*) and asterisks mark activated oocytes (endomitotic). Proximal is to the left. Alleles, *fog-1(q185)*, *daf-18(nr2037)*, *par-4(it57)*, *aak-1(tm1944)*, *par-5(it55)*. Scale bar, 20 μM.
(TIF)

S3 Fig Effects of *par-4(lf)* and *let-60(gf)* on GSC proliferation in *daf-2* mutants. (**A**) The GSC MIs of A1 hermaphrodites of the indicated genotypes/conditions were determined. An asterisk indicates statistical significance (P<0.05; ANOVA with Tukey HSD multiple comparisons) to all other samples. n, 18–34. (**B**) The number of GSC per animal at the dauer stage was evaluated as previously described [2] in animals of the indicated genotypes. P>0.05; two-tailed t-test. n, 25–37. (**A-B**) Dots mark averages, open boxes and brackets represent the whole sample, divided in quartiles. ns, Not significant. Alleles, *daf-2(e1370)*, *par-4(it57)*, *let-60(ga89)gf*. *qIs56* is present in the *daf-2; par-4* strain.
(TIF)

S4 Fig dpMPK-1 signal intensity profiles related to Fig 4. (**D-O**) The dpMPK-1 signal intensity in arbitrary units (A.U.) of fluorescence was background subtracted and plotted as a function of the distance across germline zone I (from early/mid-pachytene to oocytes). As *fog-2* mutants having accumulated oocytes are negative for dpMPK-1 [19,31], we used this condition as reference and considered regions of more than 100 pixels wide having signal intensities above 50 A.U. as positive for dpMPK-1 (arrows delineate zone I, except in I where dpMPK-1 is found ectopically). Refer to the materials and methods for additional information.
(TIF)

S5 Fig Additional images related to Fig 4. (**D’-P”‘**) Additional representative germ lines extruded from unmated or continually mated animals of the indicated genotypes and ages, stained with DAPI (DNA; cyan) and a monoclonal mouse anti-MAPKYT antibody labeling dpMPK-1 (green). (**P’-P”‘**) A1 fog-1 germ lines were indistinguishable from A1 fog-2 germ lines (**E’-E”**). Maximal projections in (**J”**) did not stitch properly.
(TIF)

S6 Fig Differentiated germ cell-independent effects of *mpk-1* loss-of-function on GSC proliferation. (**A-B**) Three representative A1 animals of the indicated genotypes stained with DAPI are shown. Germline tumours grow noticeably slower in the absence of *mpk-1*. Anterior is to the left and dorsal, is up. Scale bar, 50 μM. (**C**) The GSC MIs of A1 hermaphrodites of the indicated genotypes were determined. An area located around the bend of the germ line was randomly selected using the DAPI channel for MI analysis. ns, Not significant (P>0.05; two-tailed t-test). n, 25–30. (**A-C**) Alleles, *gld-3(q730)*, *nos-3(q650)*, *mpk-1(ga117)*.
(TIF)
