# Supplementary figures and images for: DAF-18/PTEN signals through AAK-1/AMPK to inhibit MPK-1/MAPK in feedback control of germline stem cell proliferation
Source: PLoS Genet. 2017 Apr 14;13(4):e1006738. doi: 10.1371/journal.pgen.1006738 (PMC5409174; doi:10.1371/journal.pgen.1006738)

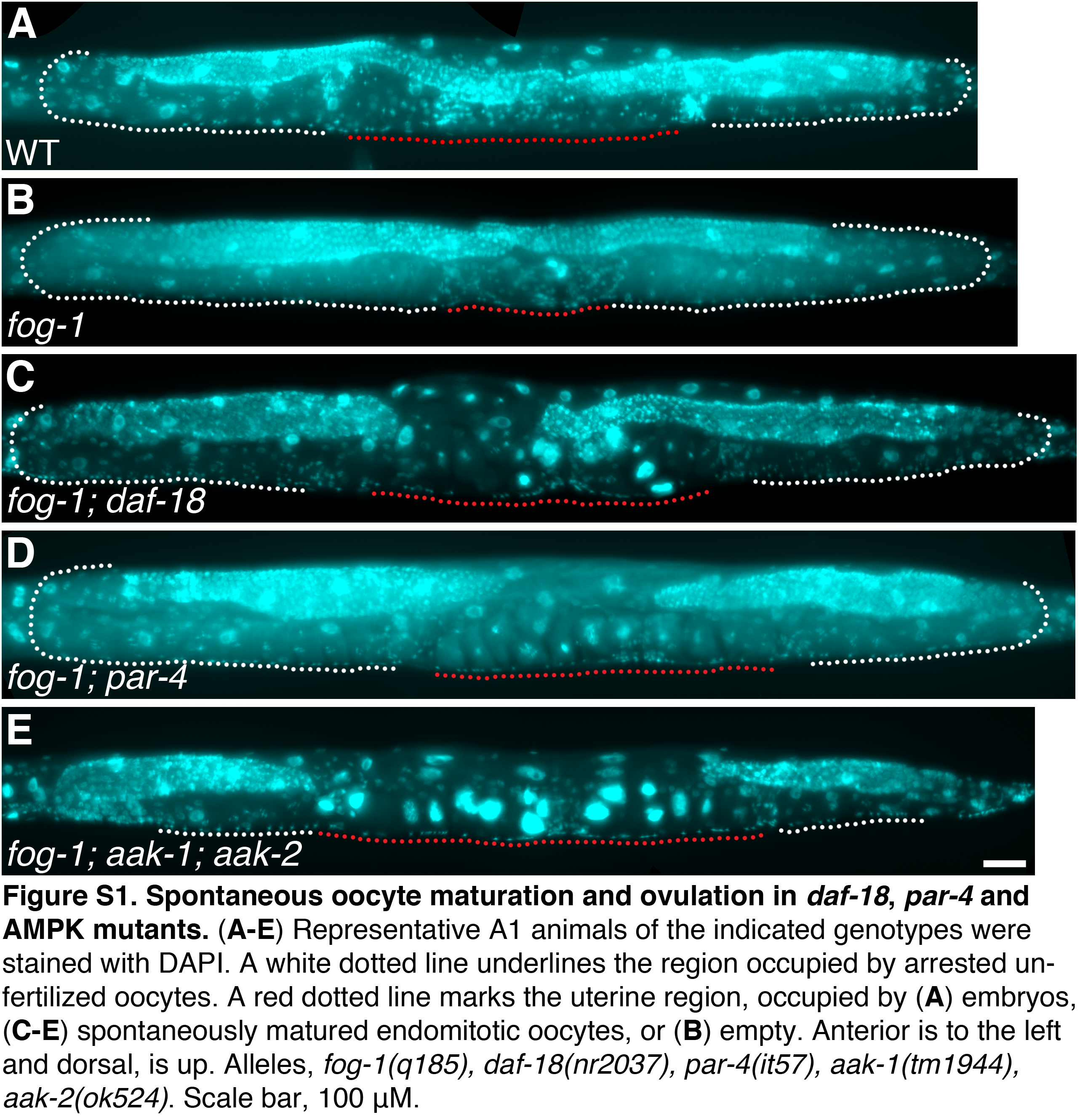

Supplement: S1 Fig — (A-E) Representative A1 animals of the indicated genotypes were stained with DAPI. A white dotted line underlines the region occupied by arrested unfertilized oocytes. A red dotted line marks the uterine region, occupied by (A) embryos, (C-E) spontaneously matured endomitotic oocytes, or (B) empty. Anterior is to the left and dorsal, is up. Alleles, fog-1(q185), daf-18(nr2037), par-4(it57), aak-1(tm1944), aak-2(ok524). Scale bar, 100 μM. (TIF) [file pgen.1006738.s001.tif]

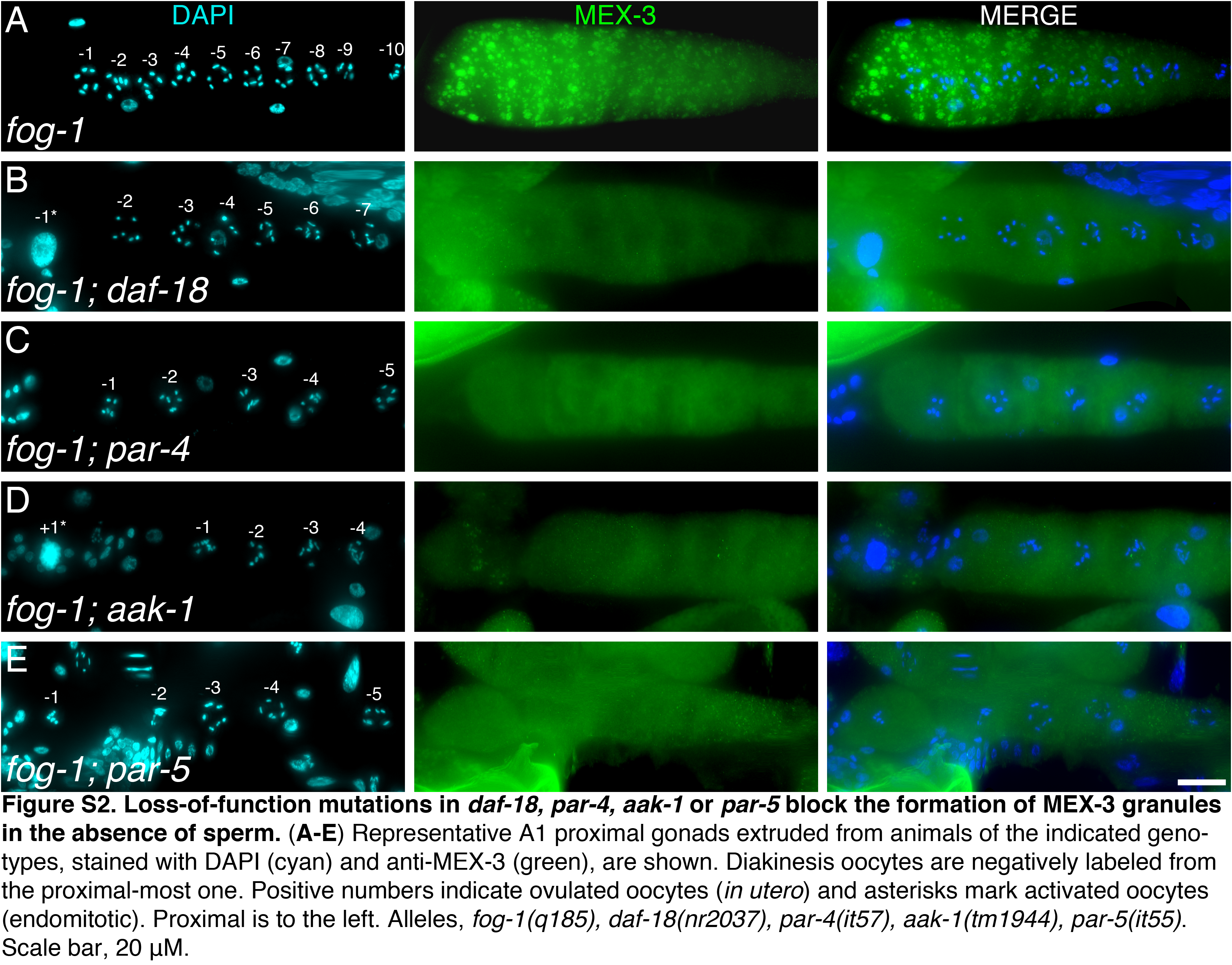

Supplement: S2 Fig — (A-E) Representative A1 proximal gonads extruded from animals of the indicated genotypes, stained with DAPI (cyan) and anti-MEX-3 (green), are shown. Diakinesis oocytes are negatively labeled from the proximal-most one. Positive numbers indicate ovulated oocytes (in utero) and asterisks mark activated oocytes (endomitotic). Proximal is to the left. Alleles, fog-1(q185), daf-18(nr2037), par-4(it57), aak-1(tm1944), par-5(it55). Scale bar, 20 μM. (TIF) [file pgen.1006738.s002.tif]

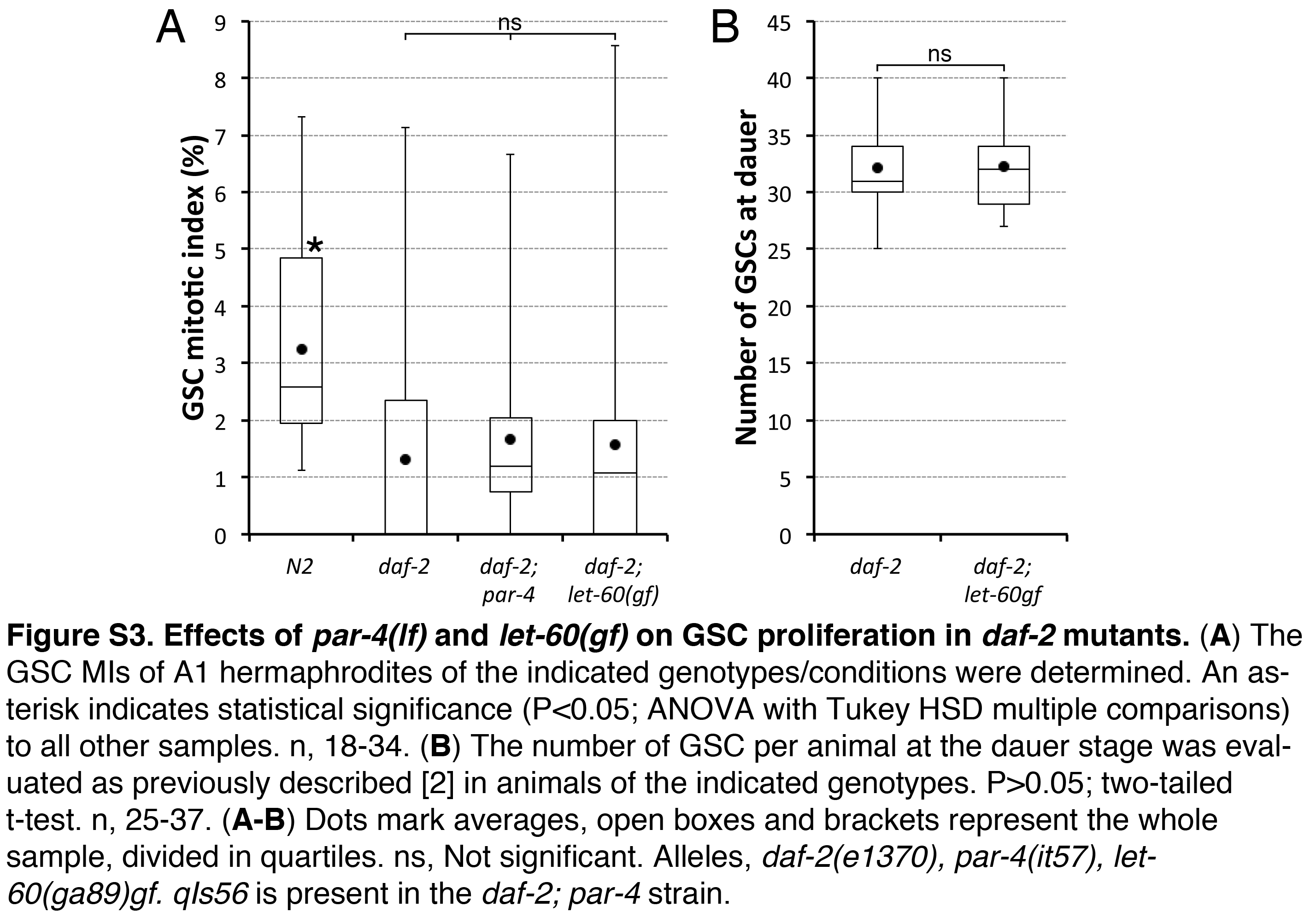

Supplement: S3 Fig — (A) The GSC MIs of A1 hermaphrodites of the indicated genotypes/conditions were determined. An asterisk indicates statistical significance (P<0.05; ANOVA with Tukey HSD multiple comparisons) to all other samples. n, 18–34. (B) The number of GSC per animal at the dauer stage was evaluated as previously described [2] in animals of the indicated genotypes. P>0.05; two-tailed t-test. n, 25–37. (A-B) Dots mark averages, open boxes and brackets represent the whole sample, divided in quartiles. ns, Not significant. Alleles, daf-2(e1370), par-4(it57), let-60(ga89)gf. qIs56 is present in the daf-2; par-4 strain. (TIF) [file pgen.1006738.s003.tif]

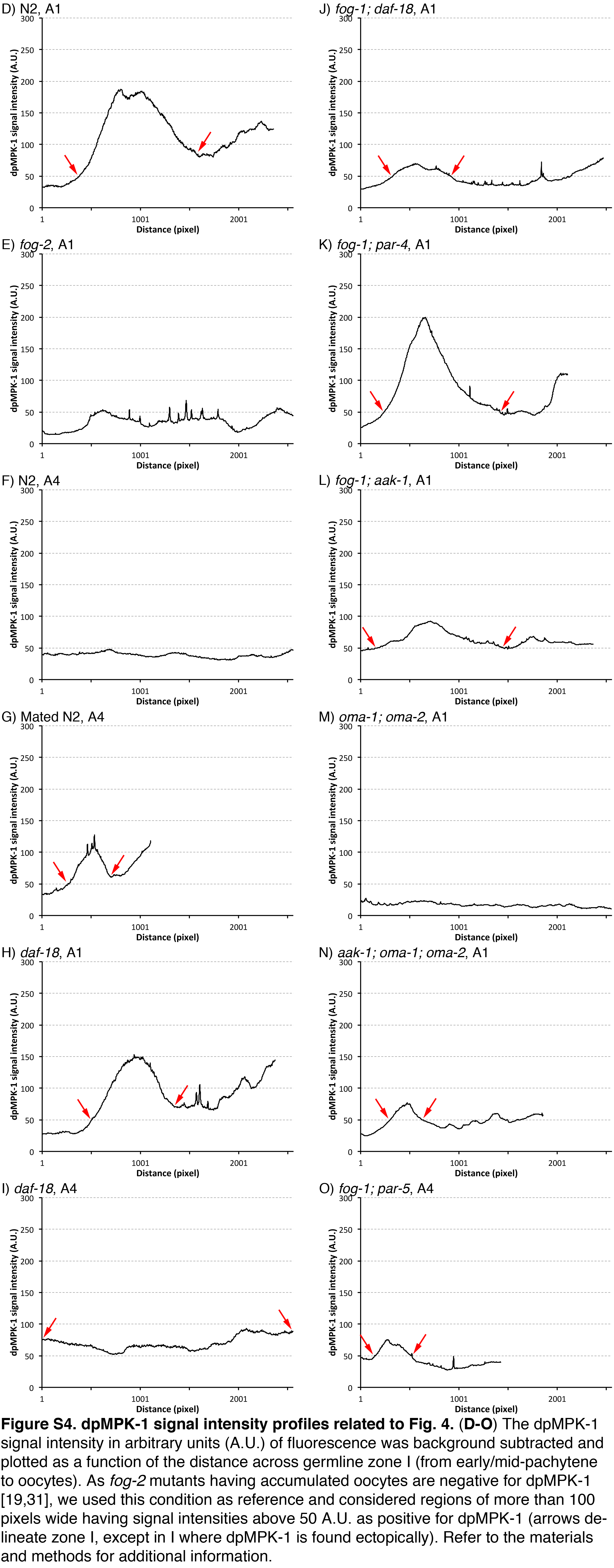

Supplement: S4 Fig — (D-O) The dpMPK-1 signal intensity in arbitrary units (A.U.) of fluorescence was background subtracted and plotted as a function of the distance across germline zone I (from early/mid-pachytene to oocytes). As fog-2 mutants having accumulated oocytes are negative for dpMPK-1 [19,31], we used this condition as reference and considered regions of more than 100 pixels wide having signal intensities above 50 A.U. as positive for dpMPK-1 (arrows delineate zone I, except in I where dpMPK-1 is found ectopically). Refer to the materials and methods for additional information. (TIF) [file pgen.1006738.s004.tif]

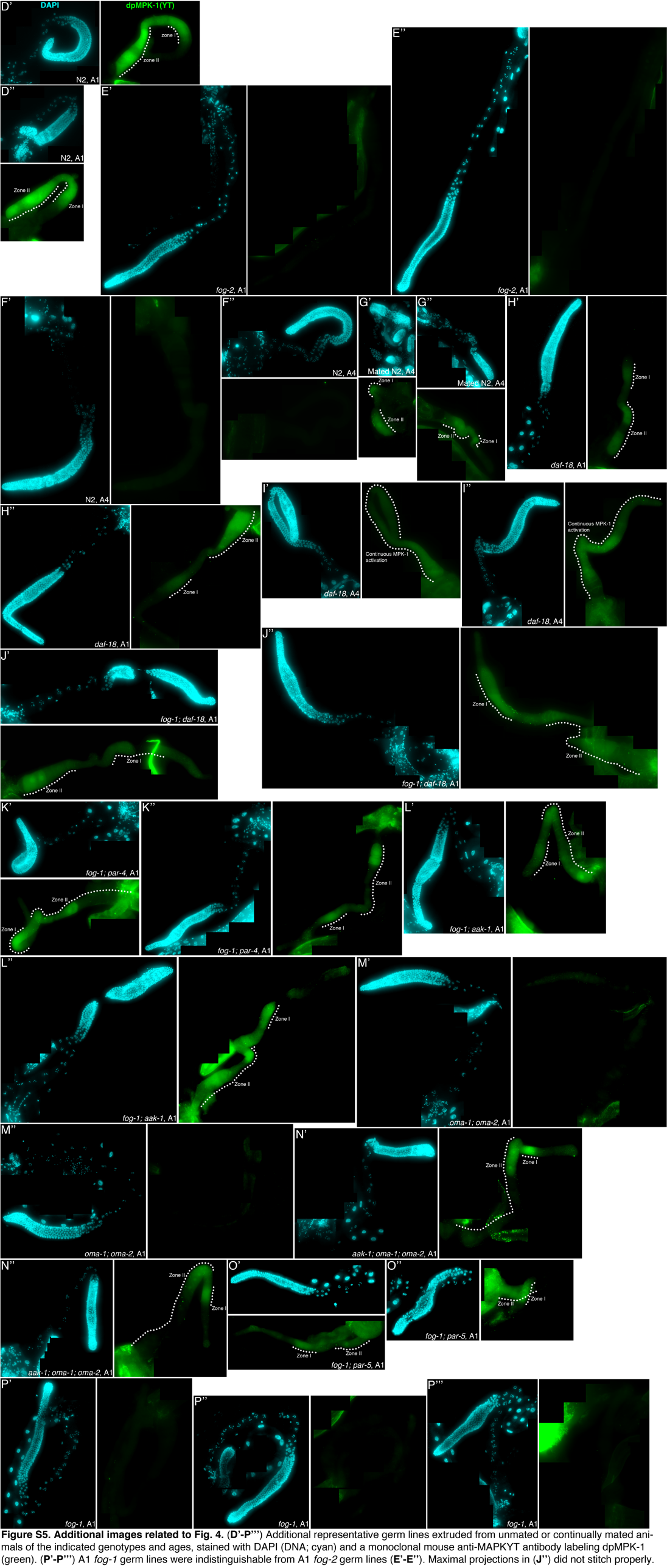

Supplement: S5 Fig — (D’-P”‘) Additional representative germ lines extruded from unmated or continually mated animals of the indicated genotypes and ages, stained with DAPI (DNA; cyan) and a monoclonal mouse anti-MAPKYT antibody labeling dpMPK-1 (green). (P’-P”‘) A1 fog-1 germ lines were indistinguishable from A1 fog-2 germ lines (E’-E”). Maximal projections in (J”) did not stitch properly. (TIF) [file pgen.1006738.s005.tif]

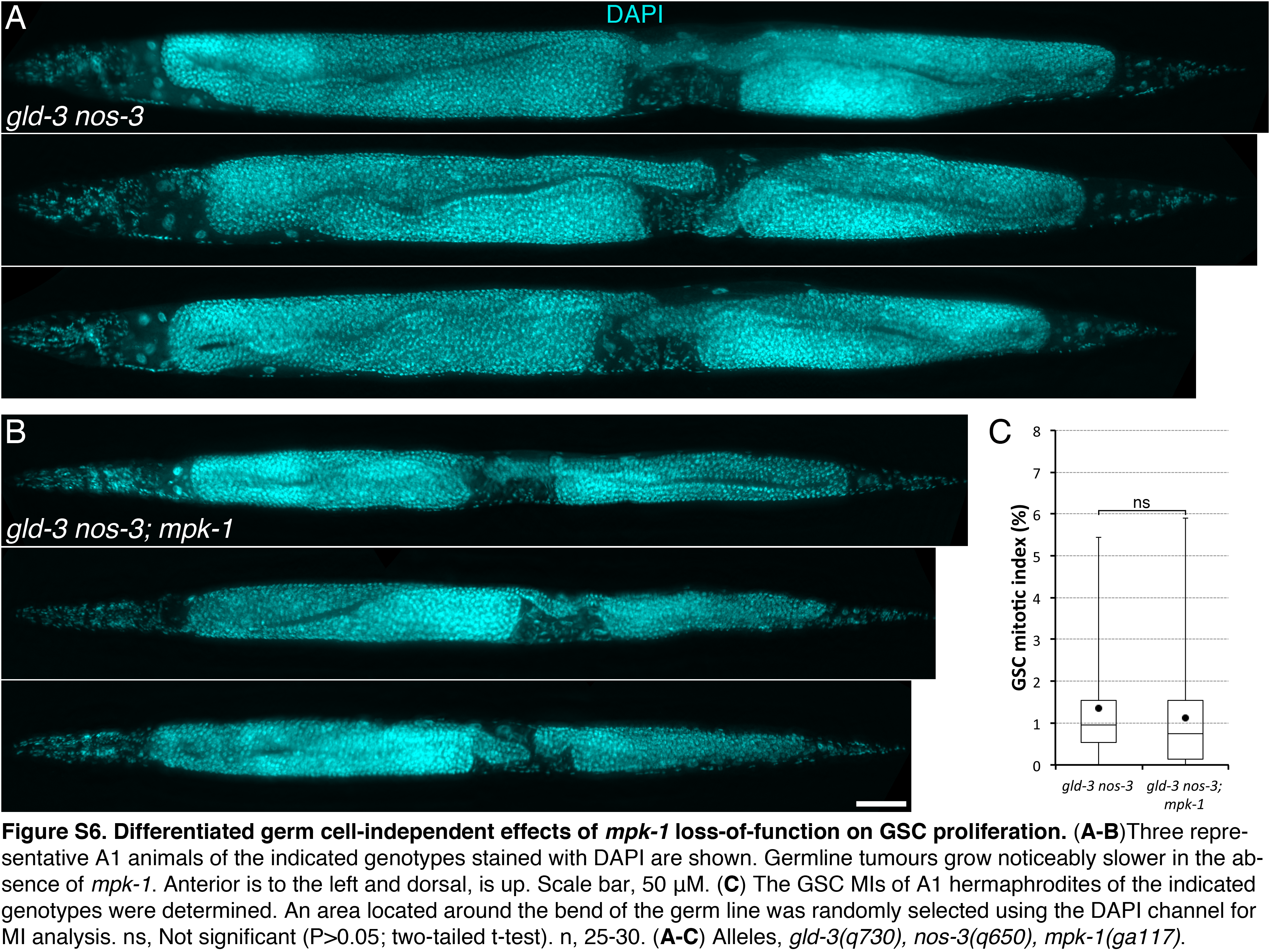

Supplement: S6 Fig — (A-B) Three representative A1 animals of the indicated genotypes stained with DAPI are shown. Germline tumours grow noticeably slower in the absence of mpk-1. Anterior is to the left and dorsal, is up. Scale bar, 50 μM. (C) The GSC MIs of A1 hermaphrodites of the indicated genotypes were determined. An area located around the bend of the germ line was randomly selected using the DAPI channel for MI analysis. ns, Not significant (P>0.05; two-tailed t-test). n, 25–30. (A-C) Alleles, gld-3(q730), nos-3(q650), mpk-1(ga117). (TIF) [file pgen.1006738.s006.tif]
